# Crankshaft high cycle bending fatigue test method based on the combined extended Kalman filtering algorithm and different failure criterion parameters

**DOI:** 10.1371/journal.pone.0309759

**Published:** 2025-02-03

**Authors:** Zhang Wei, Yan Ruidong, Songsong Sun, Gong Xiaolin, Liu Zhentao

**Affiliations:** 1 Power Machinery & Vehicular Engineering Institute, Zhejiang University, Hangzhou, Zhejiang, China; 2 College of Automobile and Traffic Engineering, Nanjing Forestry University, Nanjing, Jiangsu, China; The University of British Columbia, AUSTRALIA

## Abstract

In this paper, an accelerated bending fatigue test method for the crankshaft was proposed based on the combination of the extended Kalman filtering algorithm method (EKF) and different failure criterion parameters. First the intrinsic frequency of the system and the fatigue crack depth were chosen to be the failure criterion parameters based on the test regulation and the theory of fracture mechanics. Then the extended Kalman filtering method was adopted in predicting the remaining high cycle fatigue life of the crankshaft during the experiment process. Finally the predicted fatigue life was selected to replace the actual test results for the key fatigue property parameter analysis. Based on this research, the time duration of the test was reduced obviously without affecting the key analysis parameters obviously. In addition, compared with the intrinsic frequency of the system, the fatigue crack depth is more suitable to be selected as the failure criterion to provide more reasonable enhancing effect. The main conclusions draw from this paper can provide some theoretical guidance for the design and manufacturing process of the part.

## 1. Introduction

Nowadays, automobile is one of the most important and common traffic vehicles in our daily life [1, 2]. Being the heart of this equipment, the engine provides continuous power output during the working period. As a result of this, large enough strength is the key parameter in guaranteeing the normal operation of the equipment, especially for the parts belongs to the power assembly system such as the crankshafts.

In terms of this issue, relevant studies were carried out by the experts around the world. According to the purpose of research, these studies can be divided into two kinds. The first kind aims at researching the fatigue damage mechanism of the part to improve the processing technic. Among these Calderon found that the fretting fatigue damage mechanism of the selected crankshaft may be attributed to not only one reason, but including three factors: the improper surface heat treatment, the weakness of the material itself, and the mounting error of the bushing and bearings [3]. Hosseini proposed a fatigue crack detection method based on the acoustic emission entropy value, which is benefit for reducing the amount of data and achieving the goal of real-time monitoring [4]. Wang Yanping researched the fatigue damage mechanism of a steel crankshaft, the results showed that metallographic structure property and the stress concentration caused by the fillet are the main reasons for this phenomenon [5]. Dong Jie applied the Constructure-Kinematics-Materials-Craftsmanship (CKMC) method in analyzing the fatigue fracture surface of the selected crankshaft. The main reasons of the fracture according to this research were material defects and lack of nitrided layer at fillet [6]. Aliakbari analyzed the unusual fatigue damage feature of the selected crankshaft and discovered that the fatigue crack initiated at the lubrication hole, and compared with the stress concentration, the unqualified material property and low installation accuracy are more important in resulting the fracture [7]. For the second type, the most important thing is to predict the fatigue property of the given crankshaft accurately. Among these Qin researched the fatigue property of the crankshaft treated by the quenching process based on the critical plane approach and the residual stress field. The proposed model could fit the accuracy demands [8]. Gomes adopted the Soderberg multi-axial fatigue criterion in researching the crankshaft fatigue process, in this way corresponding optimum structural design process was carried out [9]. Leitener applied the Spagnoli fatigue model in analyzing the multi-axially loaded crankshaft fatigue property, which can accurately predict the fatigue strength of the part, as well as the fatigue crack angle [10]. Bulut analyzed the load history of the crankshaft during the working period, based on this a new fatigue safety factor can be proposed to evaluate the fatigue property more comprehensively [11]. Liu researched the relationship between the thermodynamic entropy generation rate and cumulative entropy and fatigue performance of metal components during the fatigue loading period, based on which the fatigue life prediction model based on the thermodynamic entropy of metals was proposed and could be applied in the real-time assessment of fatigue damage of metal components [12].

At present, most of the fatigue property research results should be checked through corresponding fatigue experiment. For the crankshaft, the most commonly used test method is the resonant bending fatigue test [13]. The fundamental of the equipment is that the dynamic load applied on the specimen is amplified because of the resonance effect of the test system. In this way the increase of the load frequency during the experiment can be achieved to provide high enough load for the large-scale components such as the crankshaft. As a result of this, the test duration based on this equipment is obviously shorter than that of the other type of fatigue test machines such as the hydraulic test system (usually no more than 30%). In recent years, the equipment is usually controlled through computer with predetermined program, which can ensure the accuracy of the load applied on it, as well as the failure criterion [14, 15]. The load condition provided by such test bench is quite near to that of the engine during the working period, which can be the basic of analyzing the fatigue property. While the type of the test is high cycle fatigue, which will certainly result in relatively longer period, as well as higher cost. In addition, according to the theory of fatigue and reliability, the fatigue test results usually show obvious dispersion property, especially for the high cycle cases [16, 17]. As a result of this, the high cycle fatigue test usually requires large test sample to analyze the distribution property of the fatigue strength and corresponding statistical analysis is also indispensable. Meanwhile, the experimental cost will rise again, which makes the quicker determination of the fatigue property of a given type of crankshaft becomes important. At present, several remaining life prediction approached such as the Kalman filtering algorithm methods has been carried out in actual engineering. However, the research objects in these articles are usually the operating life of the battery and some other similar components [18, 19], the application of these approaches in residual fatigue life prediction is relatively less reported in recent years.

In this paper, a new accelerated fatigue test method for the crankshaft was invented to reduce the test cost. First different kind of fatigue failure decision parameters were defined according to the given experiment standard and the theory of fracture mechanics. Then the extended Kalman filtering algorithm method was chosen to be the tool of predicting the remaining fatigue life of the crankshaft during the loading procedure. Finally the statistical analysis results of the fatigue limit load were carried out based on the predictions. The results showed that compared with the previous applied methods, the combined method proposed in this article can predict the remaining fatigue life more accurately to provide nearly the same experiment results. In addition, among the two failure decision parameters, the fatigue crack depth is more suitable on account of the more effective timesaving results, thus makes it worth adopting in the crankshaft manufacturing industry.

## 2. Method

### 2.1 The experiment method and equipment

**Fig 1:**
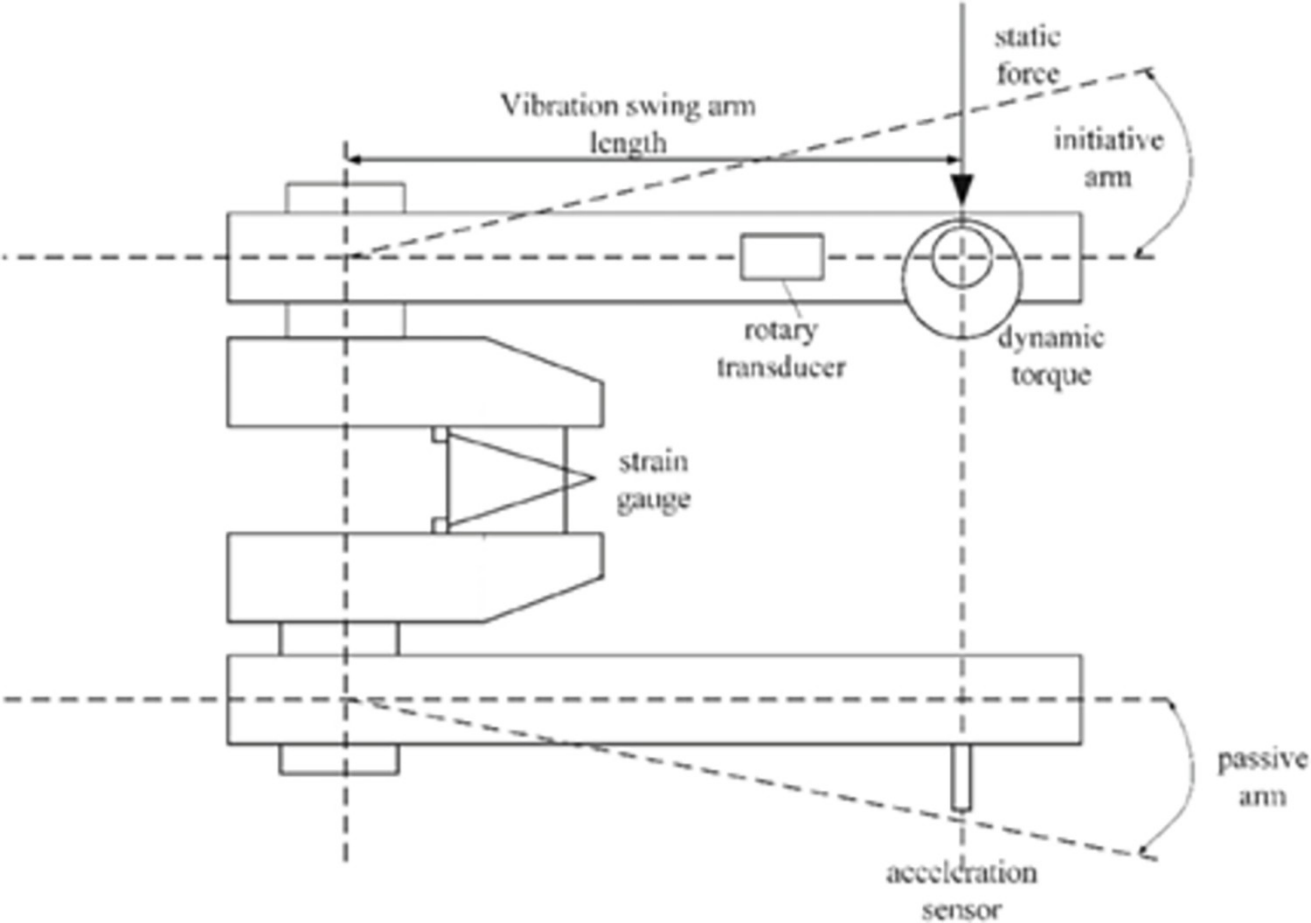
The experiment bench for the crankshaft bending fatigue test.

At present, the bending fatigue test of the crankshaft is usually carried out on the platform from the resonant bending fatigue test bench. Fig 1 shows the major structure of this test bench, among which the main parts are the swing arms (including the master arm and the driven arm), the shock excitation electric engine, and the sensors stuck to the surface of the crankshaft and the arms. During the experiment process, continual alternating bending moment is applied on the crankshaft until it breaks. Meanwhile, the degree of injury can be monitored indirectly based on the information provided by the sensors [20, 21].

**Fig 2:**
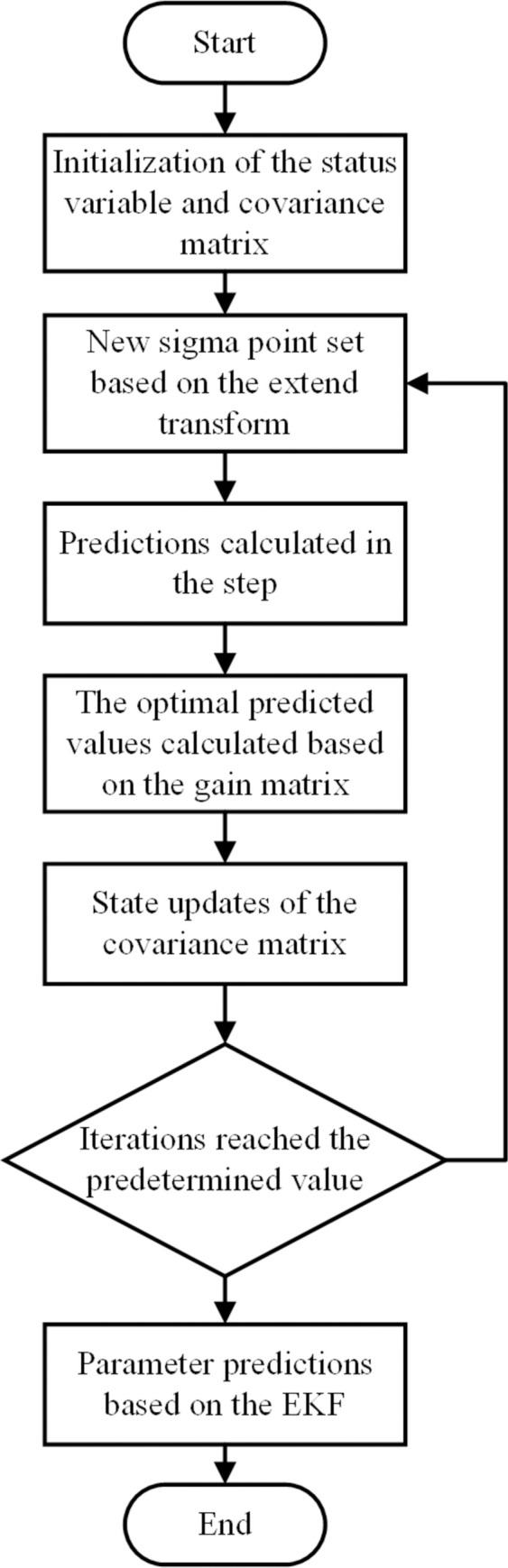
The process of the whole crankshaft bending fatigue experiment.

At present, for the industrial experiment, one of the most important things is to guarantee the accuracy of the parameter through standard calibration [22]. According to the experiment standard demands, the whole process of the experiment can be divided into three steps. The first step is the static calibration step, among which the crankshaft is clamped in the test bench and strain gaps are stick on the fillet. Then an increasing static bending load is applied on the crankpin and the value of the strain is recorded. In this way the relationship between the predetermined static bending moment and the measured strain can be obtained. The second step is the dynamic calibration step, during which the shock excitation electric engine gradually rotates more and more quickly to generate an increasing dynamic bending moment on the crankpin, and the amplitude of the moment can be measured indirectly by monitoring the value of the recorded strain. Based on this the relationship between the rotate speed and the amplitude of the alternating bending moment can be determined. In other words, the load amplitude of the bending moment during the experiment process can be controlled by adjusting the rotate speed of the electric engine, which is easy to be achieved. In the final step, the load amplitude in different experiment cases and the sample size of the whole experiment is determined according to the experiment standard to provide the corresponding load-life relationship for the statistical analysis. In this way the fatigue limit load of the crankshaft under the determined fatigue life and survival rate can be determined. The detailed information of the whole process is shown in Fig 2.

### 2.2 The fatigue failure criterion parameter determination method

**Fig 3:**
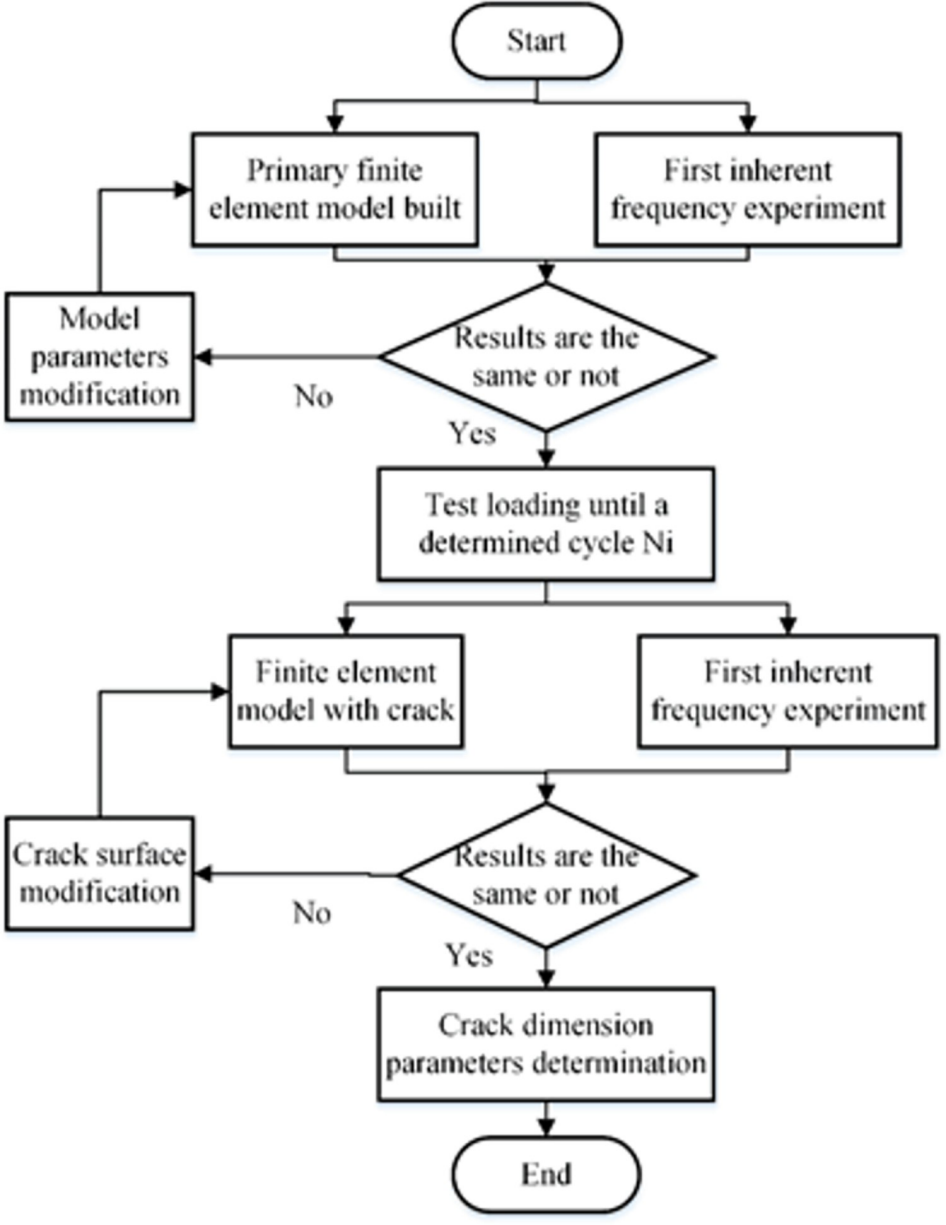
The fatigue crack determination method.

For the fatigue test experiment, one of the most important factors is the fatigue failure criterion parameter. At present, the commonly used ASTM standards have been widely applied in the bending fatigue test of the materials [23, 24]. While for the large-scale components such as the crankshaft, if the rule is too strict, the fatigue test results will led to redundancy design. If the rule is too loose, the safety and reliability of the test results can’t be guaranteed. At present, the industrial standard in this field is built based on the dynamic response property of the component. This parameter is easy to be measured during the experiment process. On the other hand, the material of the crankshaft is the high strength alloy steel. According to the theory of fracture mechanics, for the components made by this type of material, usually obvious fatigue crack growth and propagation process can be discovered during the fatigue damage process. Thus the fatigue damage process can be monitored indirectly by the fatigue crack length. According to our previous study, the length of the fatigue crack can be indirectly by a combined finite element and dynamic response property [25]. The detailed process of the method is shown in Fig 3.

### 2.3 The residual fatigue life prediction method

According to the experiment demand, in each test case, the specified alternating bending moment is applied on the crankshaft throughout the test process until it breaks. Thus a possible method to quicken this process is to predict the remaining fatigue life before the final break happens. At present, the remaining life prediction research has been widely promoted in some important industry component such as the lithium battery, and various kinds of models and approaches were proposed with satisfactory precision of prediction [26–28]. Meanwhile, for the fatigue damage process of the metal components such as the crankshafts, an important feature is that the accumulation rate is not steady throughout the whole process, but shows apparent nonlinear characteristic. According to previous study, the extended Kalman filtering algorithm is considered to be valuable in this application situation. The theoretical basis of this method can be expressed as:

A set of Sigma sampling points is used to describe the Gaussian distribution of random variables, and then the weighted statistical linear regression technique is used to approximate the posterior mean and variance of the nonlinear function by passing the nonlinear function.

According to the definition of this method, a nonlinear system can be defined as:

$$\begin{array}{c} \delta (k+1|k)=\delta (k)+W(k)\\ \delta (k)={\left[b(k)\;c(k)\;d(k)\right]}^{T} \end{array}$$

Where δ(k+1|k) is the observed value of the kth alternating load node, and δ(k) is the estimated value of the previous alternating load node. According to the state estimate and the corresponding system error covariance matrix, the sigma point on this time node can be determined, and the result can be expressed as:

$$\begin{array}{c} \delta^{\left(0\right)}=\bar{\delta },i=0\\ \delta^{\left(i\right)}=\bar{\delta }+{\left(\sqrt{(n+\lambda )P}\right)}_{i},i=1∼n\\ \delta^{\left(i\right)}=\bar{\delta }-{\left(\sqrt{(n+\lambda )P}\right)}_{i},i=n+1∼2n \end{array}$$

where λ is the scale factor and n is the dimension of the state variable. The weight coefficient of each point is defined as:

$$\begin{array}{c} \omega_{m}^{\left(0\right)}=\frac{\lambda }{n+\lambda }\\ \omega_{c}^{\left(0\right)}=\frac{\lambda }{n+\lambda }+(1-\alpha^{2}+\beta )\\ \omega_{m}^{\left(i\right)}=\omega_{c}^{\left(i\right)}=\frac{\lambda }{2(n+\lambda )},i=1∼2n \end{array}$$

Where *i* is the number of the sigma points, *ωm* and *ωc* are the weight coefficients of the mean value and the variance respectively. By combining these two equations above, the non- linear mapping of these sigma points can be expressed as:

$$\delta^{\left(i\right)}(k+1|k)=f[k,\delta^{\left(i\right)}(k|k)]=\sum_{i=0}^{2n}\omega^{\left(i\right)}\delta^{\left(i\right)}(k+1|k)$$

$$S(k+1|k)=\sum_{i=0}^{2n}\omega^{\left(i\right)}[\hat{\delta }(k+1|k)-\delta^{\left(i\right)}(k+1|k)]{\left[\hat{\delta }(k+1|k)-\delta^{\left(i\right)}(k+1|k)\right]}^{T}$$

As shown in the formula above, *δ*(*i*)(*k*+1|*k*) is the predicted value and *S*(*k*+1|*k*) is the covariance matrix. Based on these parameters, the new sigma point set can be proposed with the help of the unscented transform method, the result can be expressed as:

$$\delta^{\left(i\right)}(k+1|k)=[\hat{\delta }(k+1|k)\hat{\delta }(k+1|k)+\sqrt{\left(n+\lambda )S(k+1|k\right)}\hat{\delta }(k+1|k)-\sqrt{\left(n+\lambda )S(k+1|k\right)}]$$

By taking these new sigma points into the observation and prediction equations, the mean value and the covariance of the predictions can be determined as:

$$S_{z_{k}z_{k}}=\sum_{i=0}^{2n}\omega^{\left(i\right)}[Z^{\left(i\right)}(k+1|k)-\bar{\zeta }(k+1|k)]{\left[\zeta^{\left(i\right)}(k+1|k)-\bar{\zeta }(k+1|k)\right]}^{T}+R$$

$$S_{x_{k}z_{k}}=\sum_{i=0}^{2n}\omega^{\left(i\right)}[\delta^{\left(i\right)}(k+1|k)-\bar{\zeta }(k+1|k)]{\left[\delta^{\left(i\right)}(k+1|k)-\bar{\zeta }(k+1|k)\right]}^{T}$$

Finally the Kalman gain matrix and the system status and covariance updates can be deter- mined as:

$$K(k+1)=S_{x_{k}z_{k}}S_{z_{k}z_{k}}^{-1}$$

$$\hat{\delta }(k+1|k+1)=\hat{\delta }(k+1|k)+K(k+1)[\zeta (k+1)-\hat{\zeta }(k+1|k)]$$

$$S(k+1|k+1)=S(k+1|k)-K(k+1)S_{z_{k}z_{k}}K^{T}(k+1)$$

**Fig 4:**
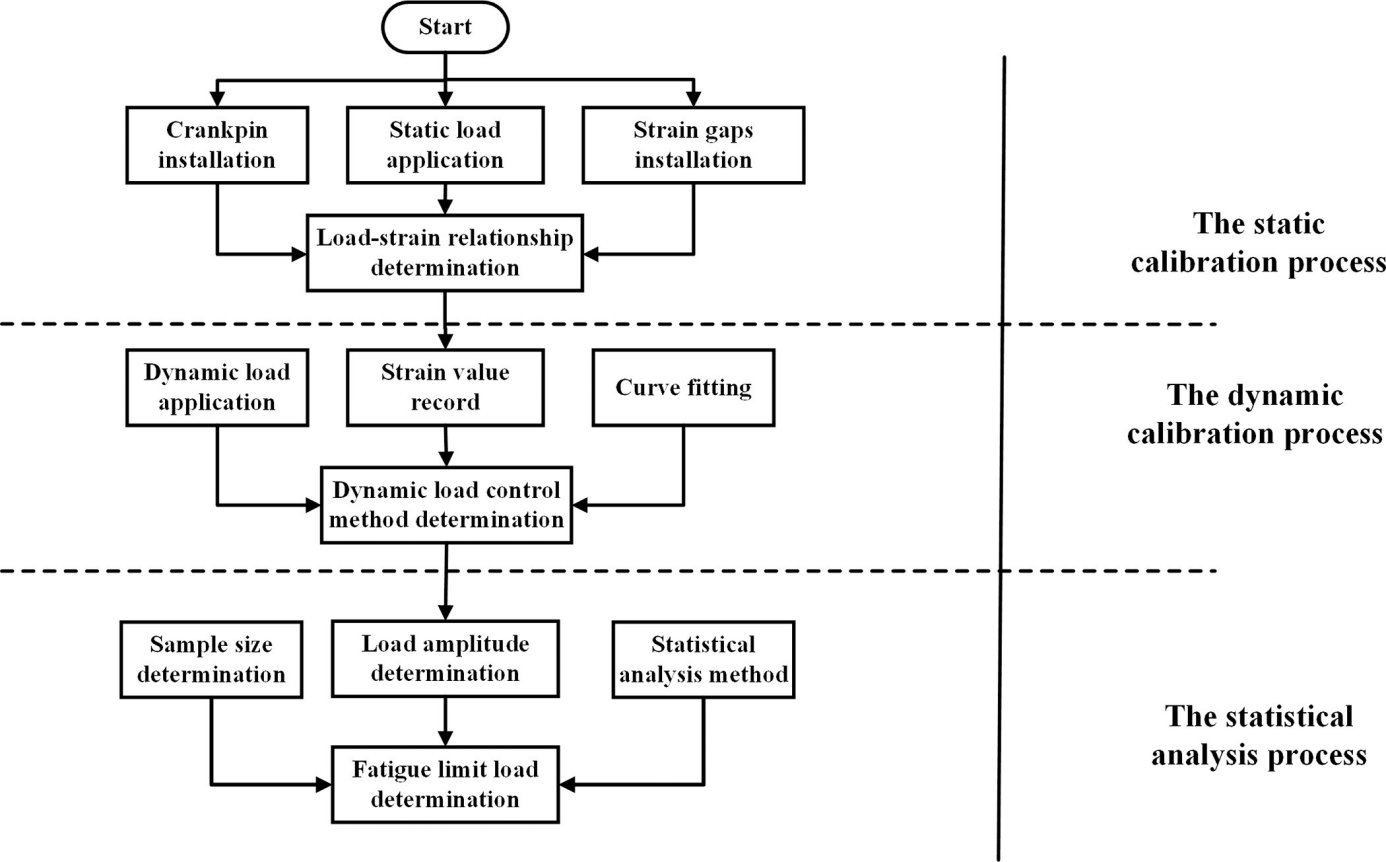
The prediction process of the residual fatigue life based on the EKF.

Based on this approach, the residual fatigue life prediction can be proposed. The detailed process of the method is shown in Fig 4.

### 2.4 The statistical analysis method

**Fig 5:**
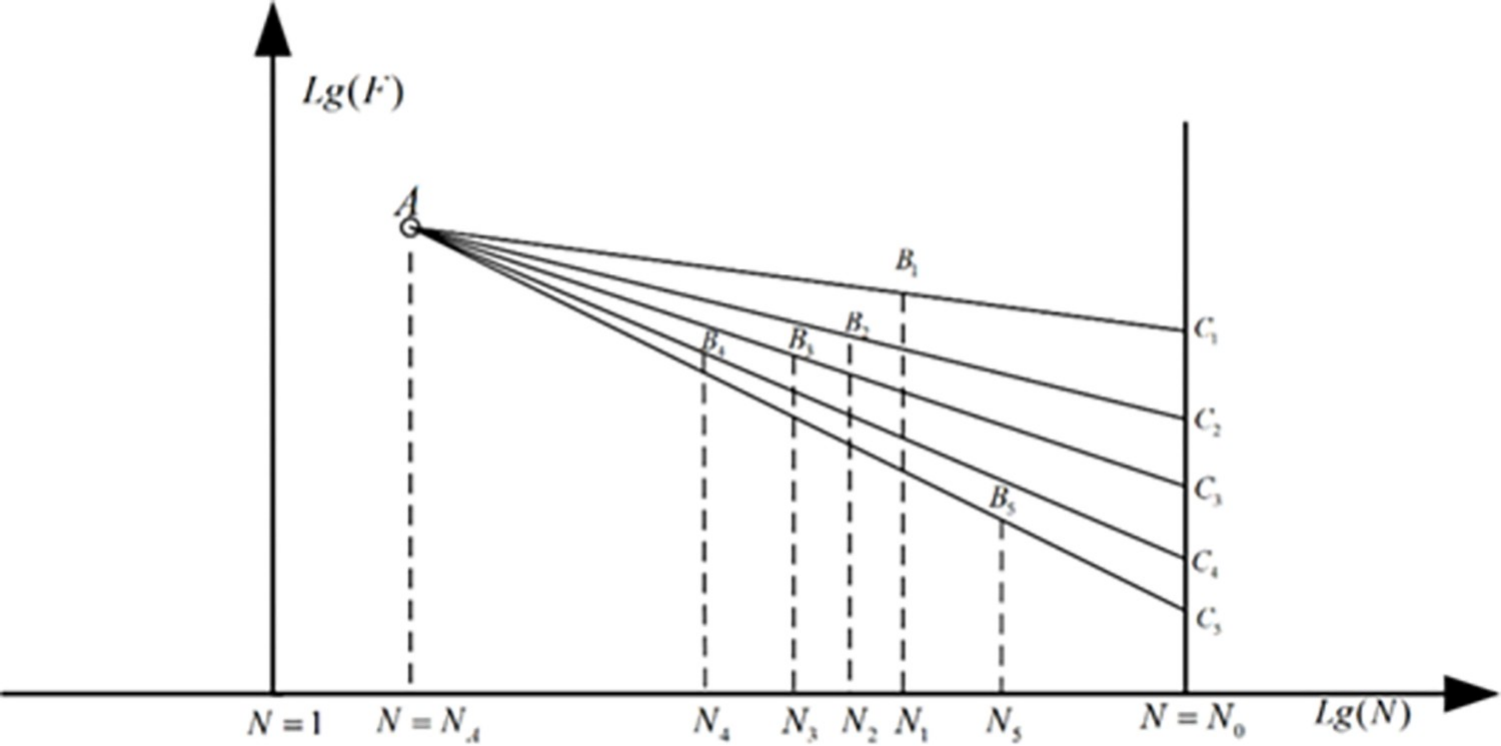
The stress-life relationship expressed through log-log coordinate.

For the fatigue experiment results, one of the most important features is the great dispersion, which makes the statistical analysis absolutely indispensable. Fig 5 shows the theoretical basis of this method, among which the point A is the stress amplitude at the low cycle fatigue life. In addition, all the S-N curves under any survival rate will cross this point [29]. As a result of this, the fatigue limit load analysis result in the ith case can be expressed as:

$$\mathrm{lg}\;C_{i}=\mathrm{lg}\;F_{A}\frac{\mathrm{lg}\;N_{0}-\mathrm{lg}\;N_{i}}{\mathrm{lg}\;N_{A}-\mathrm{lg}\;N_{i}}+\mathrm{lg}\;F_{i}\frac{\mathrm{lg}\;N_{0}-\mathrm{lg}\;N_{A}}{\mathrm{lg}\;N_{i}-\mathrm{lg}\;N_{A}}$$

where *F*_*A*_ is the load amplitude which can be determined through a least squares fit approach of the experiment data. In this way, the distribution property of the fatigue limit load at the specified limit life can be determined.

## 3. Results

### 3.1 The fracture surface analysis results

According to the research method analysis results in the previous chapter, it’s possible to carry out the accelerated fatigue test method based on the remaining fatigue life prediction process. The research object in this article is a type of crankshaft from a straight-six diesel engine. The material of the crankshaft is 42CrMo, a typical kind of high strength alloy steel. The detailed geometric structure parameters of the part is shown in Table 1.

**Table 1:** The main structural parameters of the crankshaft.

Parameter	Value
Crankpin diameter	82 *mm*
Crank width	100 *mm*
Main journal diameter	29 *mm*
Fillet	5 *mm*
Overlap	26 *mm*

**Fig 6:**
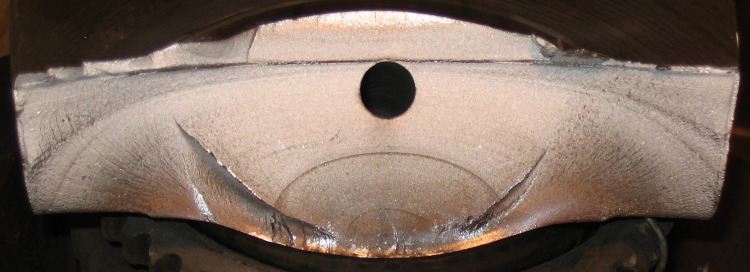
The fracture surface of the crankshaft.

Based on the research method mentioned above, it’s possible to carry out the fatigue research of the crankshaft. Fig 6 shows fracture surface of the crankshaft, which can be determined to be the typical conchoidal fatigue striations and fatigue steps and secondary cracks are observed on some of the fractures. Combining this feature and the experimental data, the fatigue model of the crankshaft can be the high cycle fatigue. In addition, the fatigue initiates from the fillet of the crankpin and gradually propagates to the fillet of the main journal. The evolution process of the fatigue crack in shown in Table 2.

**Table 2:** The structure parameter evolution process of the fatigue crack surface.

a/*mm*	b/*mm*
1	3.8
2	4.0
3	4.2
4	4.5
5	5.1
6	5.5
7	8.1
8	9.3
9	10.1
10	10.8

Based on the fatigue crack evolution process, the relationship between the fatigue crack depth and the frequency of the system can be determined. The results are shown in Table 3.

**Table 3:** The influence of the fatigue crack on the system frequency.

a/*mm*	frequency/*Hz*
0	46.2
1	46.18
2	46.14
3	46.08
4	46.04
5	45.98
6	45.86
7	45.72
8	45.56
9	45.38
10	45.19

### 3.2 The failure criterion analysis results

**Fig 7:**
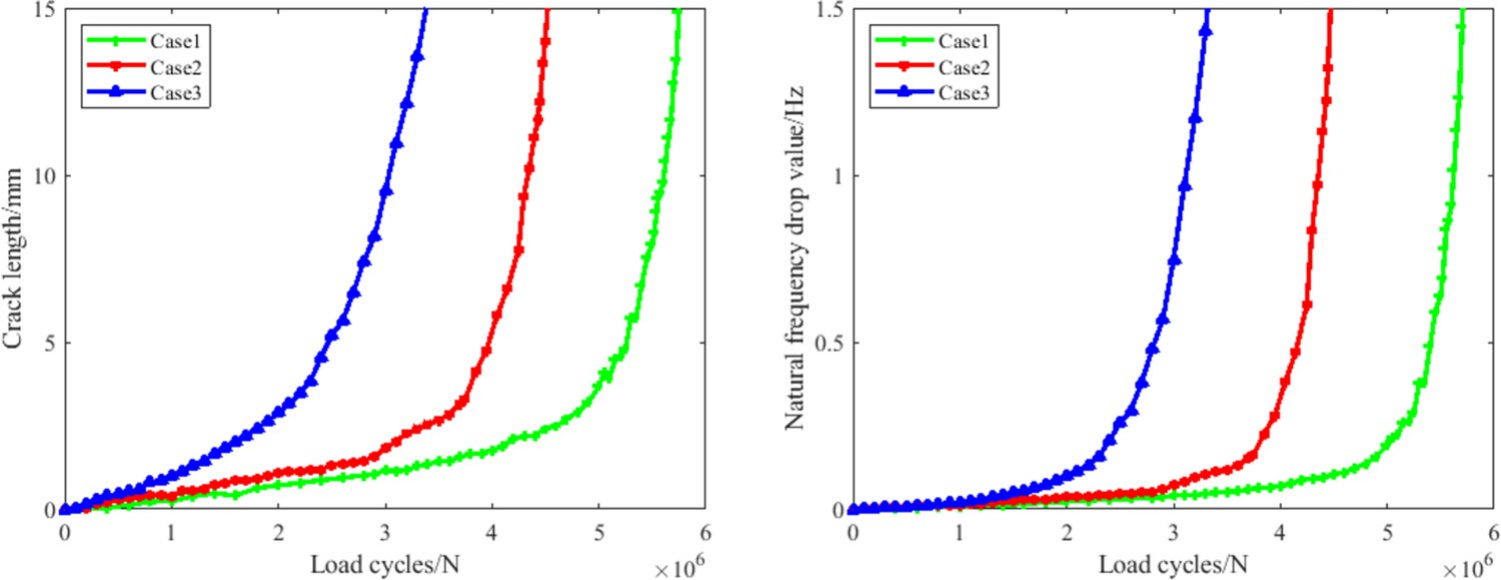
The evolution process of both failure criterion parameters (a. fatigue crack depth, b. first order inherent frequency).

In this paper, three groups of crankshafts were selected to be the candidates of our research. At present, the crankshaft fatigue failure criterions are usually achieved based on two factors: the decrement of the first order inherent frequency, and the fatigue crack depth. Fig 7 shows the fatigue damage parameter evolution process of all the three cases based on these two parameters. It can be found clearly that when the inherent frequency of the crankshaft is selected to be the failure criterion parameter, the decreased value of the parameter begins to grow more quickly in all the three cases, when it has reached 1 *Hz*. This phenomenon is in accordance with the test standard. While for the fatigue crack, an obvious feature of the evolution process is that the values of the crack growth speed is not steady. Instead the growth speed increases gradually within different stages. When the crack depth has reached 10 *mm*, the growth speed increases rapidly (the slope of the curve is nearly vertical), which means that the fatigue crack growth rate from then on becomes unsteady and will soon result in the failure. Thus this value is set to be the failure decision value.

### 3.3 Predictions based on the frequency

Based on the failure criterion parameter analysis results proposed in the previous chapter, it’s possible to carry out the corresponding remaining fatigue life prediction study, as well as the acceleration effect of the experiment. According to our previous research, the prediction approach can be divided into two main steps [30]. The first step is to train the selected prediction model based on the earlier evolution stage during the whole damage process. In this way the model parameters can be fixed for the predicting the evolution process in the further coming stage in the second step.

**Fig 8:**
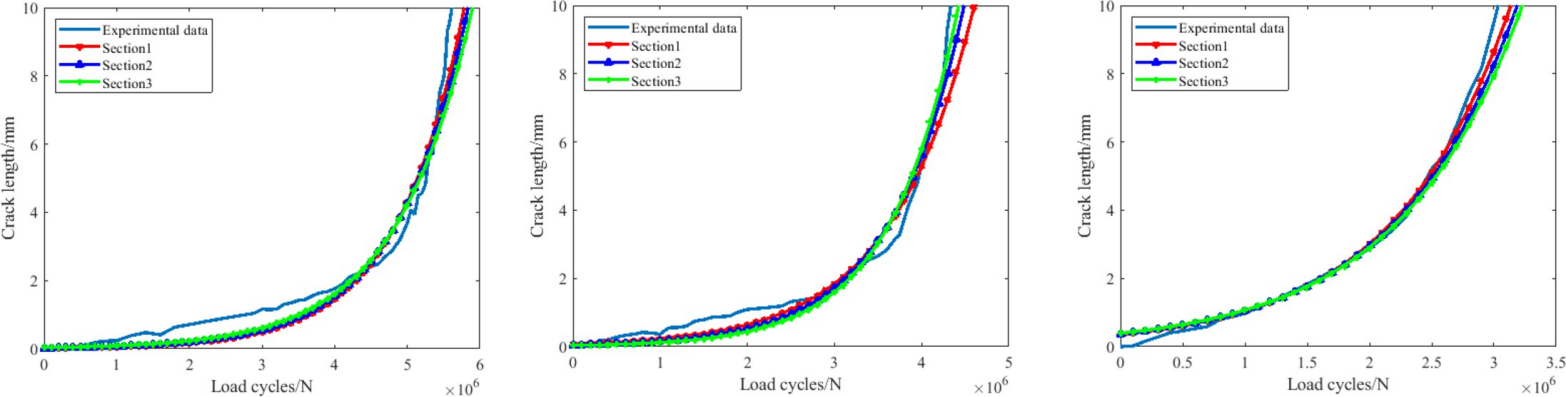
The remaining fatigue life predictions based on the frequency(case1)(case2)(case3).

Table 4 shows the detailed description of these different kinds of section definitions, among which a clear conclusion can be proposed that the smaller size the training section, the more experiment time can be saved. In addition, according to the related research, these types of section definitions can provide higher accuracy than the conventional types. The predictions of all the three groups of crankshaft based on this failure criterion parameter can be deduced. Corresponding results are shown in Fig 8.

**Table 4:** The detailed definition information of the training and prediction sections based on the first fatigue failure criterion parameter.

Training sections			Prediction section		
Serial number	From	To	Serial number	Start point	End point
Section 1	0.1 *Hz*	0.2 *Hz*	Section 1	0.2 *Hz*	1 *Hz*
Section 2	0.1 *Hz*	0.3 *Hz*	Section 2	0.3 *Hz*	1 *Hz*
Section 3	0.1 *Hz*	0.4 *Hz*	Section 3	0.4 *Hz*	1 *Hz*

As shown in Fig 8(A), it’s not hard to see that with the combination of the EKF model and all the three training sections, the remaining fatigue life of the crankshaft can be accurately predicted, the errors among these predictions are all less than 7%, which is perfectly enough for the engineering application in most situations. In Fig 8(B), similar circumstances are also evident in this selected crankshaft. The predictions are nearly the same to the actual experiment data evolution process, consequently can be absolutely taken to replace the original experiment data to achieve the goal of saving time. For the predictions of case3 in Fig 8(C), the error generated based on the first training section (about 8%) is bigger than those based on the other two (about 5% or less), still precision can be provided by this method. Therefore a primary conclusion can be drawn that this failure parameter can be useful in predicting the crankshaft remaining fatigue life with the combination of our method.

### 3.4 Predictions based on the crack depth

In order to evaluate the feasibility of the prediction method more comprehensively, the predictions based on the second failure criterion parameter (the fatigue crack depth) are also exhibited to make the comparative study. Table 5 shows the corresponding description of the different training and prediction sections based on this failure criterion parameter, which is similar to that based on the first fatigue failure parameter in removing the evolution process of the fatigue crack in the earlier stage (0–1 *mm*). In this way higher precision effect can be expected.

**Table 5:** The detailed definition information of the training and prediction sections based on the second fatigue failure criterion parameter.

Training sections			Prediction section		
Serial number	From	To	Serial number	Start point	End point
Section 1	1 *mm*	3 *mm*	Section 1	3 *mm*	10 *mm*
Section 2	1 *mm*	4 *mm*	Section 2	4 *mm*	10 *mm*
Section 3	1 *mm*	5 *mm*	Section 3	5 *mm*	10 *mm*

**Fig 9:**
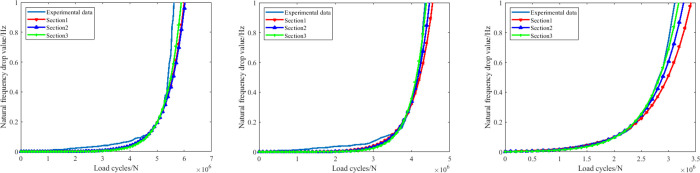
The remaining fatigue life predictions based on the fatigue crack depth(case1)(case2)(case3).

As shown in Fig 9(A), it’s clear to find that in this case, compared with the predictions based on the inherent frequency, the predictions based on the fatigue crack depth is much closer to the experiment data in all the three sections, which makes the latter parameter more applicable in this application scenarios. In Fig 9(B) and 9(C), the enhancing effect of the prediction accuracy is more obvious, the prediction results in these two cases almost coincide. By comparing these with the predictions based on the first failure criterion parameter, it’s unquestionable to announce that the fatigue crack depth is more superior in guaranteeing the prediction accuracy.

### 3.5 Timesaving and statistical analysis results

From the research conclusions proposed in the above chapter, there’s no doubt that the fatigue crack depth is superior to the inherent frequency in the prediction accuracy. On the other hand, the main purpose of the research proposed in our paper is to save experiment time, thus the percentage of the training section within the whole experiment process becomes important in achieving this goal. Table 6 shows the detailed composition of the prediction sections based on different fatigue failure criterion parameters, it’s obvious that not only the higher accuracy can be provided, the more experiment time can be saved when the fatigue crack depth is chosen. These two advantages make the parameter more suitable for the application.

**Table 6:** The percentage of the prediction section among the whole experiment process based on different fatigue failure criterion parameters.

Fatigue crack			Inherent frequency		
Section number	Case number	Percentage	Section number	Case number	Percentage
Section 1	Case 1	21.8%	Section 1	Case 1	11.1%
	Case 2	26.3%		Case 2	11.5%
	Case 3	32.3%		Case 3	22.6%
	Mean	26.8%		Mean	15.1%

According to the remaining fatigue life prediction results, when the fatigue crack depth is selected to be the failure criterion parameter, the error can be controlled within the range of no more than 7%. In our previous study, the particle filtering algorithm (PF) is also selected in predicting the residual fatigue life of the crankshaft. Table 7 shows the errors of both approaches in the predictions based on the same section (section 1 based on the fatigue crack length), from which it can be found that the errors generated by the EKF method are much lower than those generated by the PF model. This situation makes the latter approach more superior to the former in such application scenarios.

**Table 7:** The errors of the predictions based on different algorithm methods.

Group number	EKF	PF
1	3.5%	5.3%
2	5.5%	5.1%
3	3.2%	16.6%
Mean value	4.1%	9%

**Fig 10:**
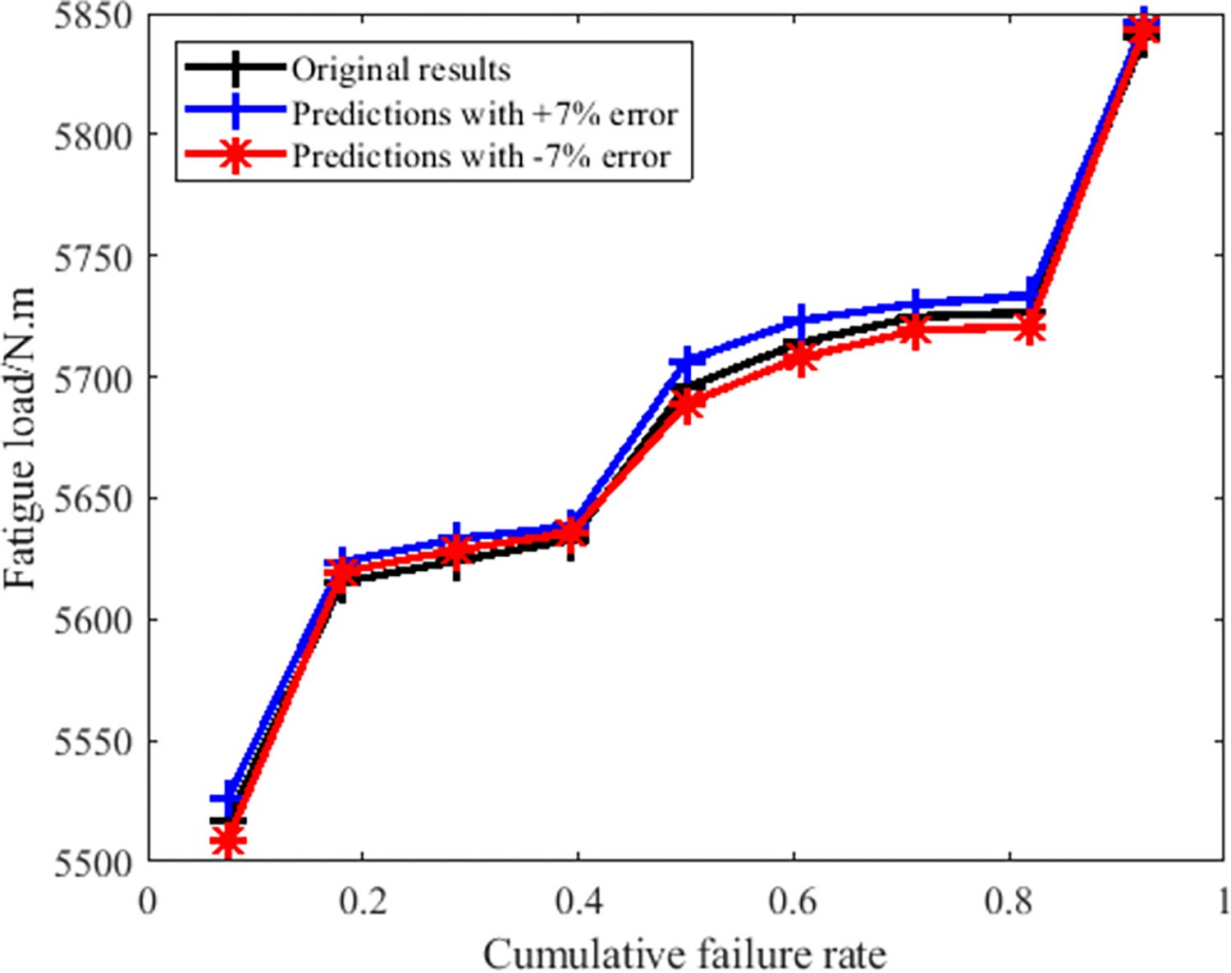
The media rank (failure rate) estimation results from different sources.

On the other hand, the key parameter in evaluating the fatigue property of the given component is usually the fatigue limit load. As a result of this, there’s need to analyze the influence of the remaining fatigue life prediction error on the final fatigue limit load. In order to make the conclusion more believable, the extreme cases were taken into consideration in this paper. Table 8 shows the actual fatigue test results of a selected type of crankshaft, among the nine groups of test results, five groups belongs to the relatively higher cycle cases (over 10^6^). So if the prediction method was applied in these cases with the maximum error output, the statistical analysis results based on them are shown in Fig 10.

**Table 8:** The fatigue test results of the crankshaft.

Load cycle	Load value/*N•m*
282484	5946
3842252	5616
590143	6014
564569	5891
5670216	5768
1200659	5867
242227	5965
4174462	5793
1945466	5842

As shown in Fig 10, it’s obvious that the fatigue limit load distribution results from the different sources are very close together, even though the maximum error has been taken into the account. Considering that the load calibration and some relative operation during the experiment process will also generate some errors, this degree of error peak value can be completely accepted.

## 4. Conclusions and further work plan

In this paper, an accelerated bending fatigue experiment method is proposed based on the extend Kalman filtering algorithm and different fatigue failure criterion parameters. First the intrinsic frequency of the system and the fatigue crack depth were chosen to be the failure criterion parameters based on the test regulation and the theory of fracture mechanics. Then the extended Kalman filtering method was adopted in predicting the remaining high cycle fatigue life of the crankshaft during the experiment process. Finally the predicted fatigue life was selected to replace the actual test results for the key fatigue property parameter analysis. The main conclusions draw from the research are as follows:

1) The combination of the extended Kalman filtering method and both failure criterion parameters (the frequency of the system and the fatigue crack length) can accurately predict the residual fatigue life of the crankshaft during the fatigue test process (the errors in all the cases are no more than 7%), which can replace the actual test results for the fatigue design of the part.

2) Among the two failure criterion parameters, the fatigue crack length is superior to the system frequency due to the more timesaving percentage (over 20% of the experiment time can be saved to reduce the cost), which is beneficial to the quick design and manufacturing of the part. In addition, compared with the particle filtering algorithm, the EKF model can provide much higher precision, these two factors make the approach proposed in this paper is more superior to the former methods.

In this paper, the residual fatigue life is predicted based on the sectional evolution process of the fatigue damage parameter during the loading period. In addition, the parameters of the selected EKF model was set according to the default values, which may lead to some errors of the predictions. In our further study, the corresponding optimized design of the EKF model parameters based on the selected algorithm method should be proposed with less training data and more accurate results, which is beneficial for the more effective timesaving design purpose.

## Supporting information

S1 File Experiment data. (DOCX)

## Abbreviations

CKMC: Constructure-Kinematics-Materials-Craftsmanship
SVM: Support Vector Machine
δ: Sigma sampling points
$\hat{\delta }$: Estimates of sigma sampling points
$\bar{\delta }$: Sample mean of the sigma points
k: At some point
λ: Scale factor
ω: Weight coefficients
α: Factor controling the distribution of sampling points
β: Factor combining moments in the variance
S: Covariance matrix
Z: Observational variable
R: Covariance matrix of Gaussian white noise
K: Kalman gain matrix
$\bar{\zeta }$: Mean of the observational variable
$\hat{\zeta }$: Estimates of the observational variable
S_A_: Stress amplitude
m: Mean value
c: Covariance
