# Supplementary material for: Crankshaft high cycle bending fatigue test method based on the combined extended Kalman filtering algorithm and different failure criterion parameters
Source: PLoS One. 2025 Feb 3;20(2):e0309759. doi: 10.1371/journal.pone.0309759 (PMC11790154; doi:10.1371/journal.pone.0309759)
Supplement: S1 File — (DOCX) [file pone.0309759.s001.docx]

| Group1 | | Group 2 | | Group3 | |
| --- | --- | --- | --- | --- | --- |
| Load cycles | Frequency | Load cycles | Frequency | Load cycles | Frequency |
| 0 | 46.1837676 | 0 | 46.18536441 | 0 | 46.20421327 |
| 200000 | 46.18287768 | 100000 | 46.18535903 | 100000 | 46.2039167 |
| 400000 | 46.18260909 | 200000 | 46.18455358 | 200000 | 46.20163402 |
| 600000 | 46.17997484 | 300000 | 46.1804031 | 300000 | 46.19952764 |
| 800000 | 46.17723969 | 400000 | 46.17965963 | 400000 | 46.19707027 |
| 1000000 | 46.17673273 | 500000 | 46.17741531 | 500000 | 46.1959948 |
| 1200000 | 46.17199096 | 600000 | 46.17567437 | 600000 | 46.19446281 |
| 1400000 | 46.16902298 | 700000 | 46.17466319 | 700000 | 46.19315221 |
| 1600000 | 46.17135413 | 800000 | 46.17288514 | 800000 | 46.18811697 |
| 1800000 | 46.16345526 | 900000 | 46.17354025 | 900000 | 46.185992 |
| 2000000 | 46.160749 | 1000000 | 46.17453272 | 1000000 | 46.18344742 |
| 2200000 | 46.15765172 | 1100000 | 46.16838078 | 1100000 | 46.17868019 |
| 2400000 | 46.15477151 | 1200000 | 46.16813215 | 1200000 | 46.17367854 |
| 2600000 | 46.15180536 | 1300000 | 46.16681632 | 1300000 | 46.16838731 |
| 2800000 | 46.14887826 | 1400000 | 46.16140089 | 1400000 | 46.16150876 |
| 2900000 | 46.14743784 | 1500000 | 46.15982572 | 1500000 | 46.15453039 |
| 3000000 | 46.1426207 | 1600000 | 46.15725384 | 1600000 | 46.14691613 |
| 3100000 | 46.14317478 | 1700000 | 46.15656521 | 1700000 | 46.13878911 |
| 3200000 | 46.14253549 | 1800000 | 46.15583844 | 1800000 | 46.12910367 |
| 3300000 | 46.13638765 | 1900000 | 46.15104996 | 1900000 | 46.11832729 |
| 3400000 | 46.1343085 | 2000000 | 46.14809533 | 2000000 | 46.10581011 |
| 3500000 | 46.13111618 | 2100000 | 46.14714756 | 2100000 | 46.09063922 |
| 3600000 | 46.12967102 | 2200000 | 46.14610523 | 2200000 | 46.07273016 |
| 3700000 | 46.12421765 | 2300000 | 46.14494839 | 2300000 | 46.04901126 |
| 3800000 | 46.12016729 | 2400000 | 46.14393839 | 2400000 | 45.99812975 |
| 3900000 | 46.11853904 | 2500000 | 46.13817318 | 2500000 | 45.94524578 |
| 4000000 | 46.11414275 | 2600000 | 46.13670458 | 2600000 | 45.90857182 |
| 4100000 | 46.10785773 | 2700000 | 46.13444156 | 2700000 | 45.82560841 |
| 4200000 | 46.09569389 | 2800000 | 46.13241069 | 2800000 | 45.72536904 |
| 4300000 | 46.0910376 | 2900000 | 46.12619598 | 2900000 | 45.63643836 |
| 4400000 | 46.09010857 | 3000000 | 46.11394099 | 3000000 | 45.45662614 |
| 4500000 | 46.07774431 | 3100000 | 46.10365952 | 3100000 | 45.23606585 |
| 4600000 | 46.07355179 | 3200000 | 46.0899637 | 3200000 | 45.0322385 |
| 4700000 | 46.05988166 | 3300000 | 46.0823423 | 3300000 | 44.77197489 |
| 4800000 | 46.04713132 | 3400000 | 46.07336385 | 3400000 | 44.40971142 |
| 4900000 | 46.02611323 | 3500000 | 46.06660294 | 3500000 | 43.82957233 |
| 5000000 | 45.99123641 | 3600000 | 46.05530693 | 3550000 | 43.49804762 |
| 5050000 | 45.95911669 | 3700000 | 46.03256642 | 3600000 | 42.37456902 |
| 5100000 | 45.97108271 | 3746000 | 46.02536637 | 3650000 | 41.30750807 |
| 5150000 | 45.92402216 | 3846000 | 45.96059419 | 0 | 46.20421327 |
| 5200000 | 45.9182904 | 3946000 | 45.90563215 | 100000 | 46.2039167 |
| 5250000 | 45.89482514 | 4046000 | 45.80369294 | 200000 | 46.20163402 |
| 5300000 | 45.80546285 | 4146000 | 45.71277655 | 300000 | 46.19952764 |
| 5350000 | 45.80393362 | 4246000 | 45.57406384 | 400000 | 46.19707027 |
| 5400000 | 45.6962173 | 4296000 | 45.34987868 | 500000 | 46.1959948 |
| 5450000 | 45.59261612 | 4346000 | 45.21616643 | 600000 | 46.19446281 |
| 5500000 | 45.54347964 | 4396000 | 45.05684672 | 700000 | 46.19315221 |
| 5520000 | 45.49246754 | 4426000 | 44.96288595 | 800000 | 46.18811697 |
| 5540000 | 45.40408205 | 4446000 | 44.86251537 | 900000 | 46.185992 |
| 5550000 | 45.34584501 | 4476000 | 44.63677341 | 1000000 | 46.18344742 |
| 5575000 | 45.31680367 | 4496000 | 44.49319065 | 1100000 | 46.17868019 |
| 5600000 | 45.26973931 | 4521000 | 44.27051842 | 1200000 | 46.17367854 |
| 5625000 | 45.16713021 | 4546000 | 43.76659208 | 1300000 | 46.16838731 |
| 5650000 | 45.04880818 | 4571000 | 43.64575228 | 1400000 | 46.16150876 |
| 5675000 | 44.95109179 | 4596000 | 43.18523138 |  |  |
| 5700000 | 44.73784362 | 4621000 | 42.52749925 |  |  |
| 5725000 | 44.59015717 | 4642000 | 40.79620494 |  |  |
| 5750000 | 44.28440924 |  |  |  |  |
| 5775000 | 44.00977128 |  |  |  |  |
| 5800000 | 43.77605571 |  |  |  |  |
| 5825000 | 43.43893216 |  |  |  |  |
| 5835000 | 43.1182145 |  |  |  |  |
| 5845000 | 42.6939832 |  |  |  |  |
| 5850000 | 42.44419665 |  |  |  |  |
| 5855000 | 41.94889547 |  |  |  |  |
| 5860000 | 41.4803855 |  |  |  |  |
|  |  |  |  |  |  |

| Group1 | | Group2 | | Group3 | |
| --- | --- | --- | --- | --- | --- |
| Load cycles | Crack depth | Load cycles | Crack depth | Load cycles | Crack depth |
| 0 | 1.82779 | 0 | 1.7693 | 0 | 1.02266 |
| 200000 | 1.85968 | 100000 | 1.7695 | 100000 | 1.14048 |
| 400000 | 1.86921 | 200000 | 1.79921 | 200000 | 1.37588 |
| 600000 | 1.96049 | 300000 | 1.94591 | 300000 | 1.47567 |
| 800000 | 2.05142 | 400000 | 1.97116 | 400000 | 1.522 |
| 1000000 | 2.06788 | 500000 | 2.04569 | 500000 | 1.67883 |
| 1200000 | 2.2165 | 600000 | 2.10187 | 600000 | 1.74109 |
| 1400000 | 2.30511 | 700000 | 2.13389 | 700000 | 1.92325 |
| 1600000 | 2.23578 | 800000 | 2.18917 | 800000 | 2.02868 |
| 1800000 | 2.46351 | 900000 | 2.16895 | 900000 | 2.32024 |
| 2000000 | 2.53724 | 1000000 | 2.13799 | 1000000 | 2.39964 |
| 2200000 | 2.61929 | 1100000 | 2.32388 | 1100000 | 2.42789 |
| 2400000 | 2.69353 | 1200000 | 2.33111 | 1200000 | 2.43526 |
| 2600000 | 2.76807 | 1300000 | 2.36904 | 1300000 | 2.47019 |
| 2800000 | 2.83986 | 1400000 | 2.51966 | 1400000 | 2.55302 |
| 2900000 | 2.87458 | 1500000 | 2.56195 | 1500000 | 2.58544 |
| 3000000 | 2.98798 | 1600000 | 2.62966 | 1600000 | 2.6365 |
| 3100000 | 2.97514 | 1700000 | 2.64752 | 1700000 | 2.67318 |
| 3200000 | 2.98995 | 1800000 | 2.66625 | 1800000 | 2.67691 |
| 3300000 | 3.12905 | 1900000 | 2.78676 | 1900000 | 2.73503 |
| 3400000 | 3.17481 | 2000000 | 2.85878 | 2000000 | 2.77215 |
| 3500000 | 3.2439 | 2100000 | 2.88153 | 2100000 | 2.82116 |
| 3600000 | 3.27473 | 2200000 | 2.90636 | 2200000 | 2.82334 |
| 3700000 | 3.38871 | 2300000 | 2.93369 | 2300000 | 2.82442 |
| 3800000 | 3.4711 | 2400000 | 2.95736 | 2400000 | 2.82442 |
| 3900000 | 3.50371 | 2500000 | 3.08925 | 2500000 | 2.86588 |
| 4000000 | 3.59037 | 2600000 | 3.12202 | 2600000 | 2.869 |
| 4100000 | 3.71096 | 2700000 | 3.1719 | 2700000 | 2.88856 |
| 4200000 | 3.93446 | 2800000 | 3.21605 | 2800000 | 2.9567 |
| 4300000 | 4.01691 | 2900000 | 3.34778 | 2900000 | 3.06516 |
| 4400000 | 4.03317 | 3000000 | 3.5943 | 3000000 | 3.06516 |
| 4500000 | 4.24396 | 3100000 | 3.7895 | 3100000 | 3.10248 |
| 4600000 | 4.31322 | 3200000 | 4.0357 | 3200000 | 3.14206 |
| 4700000 | 4.53207 | 3300000 | 4.16675 | 3300000 | 3.23754 |
| 4800000 | 4.72751 | 3400000 | 4.3163 | 3400000 | 3.24846 |
| 4900000 | 5.03389 | 3500000 | 4.42575 | 3500000 | 3.42164 |
| 5000000 | 5.50635 | 3600000 | 4.6031 | 3600000 | 3.47195 |
| 5050000 | 5.90943 | 3700000 | 4.94175 | 3700000 | 3.70822 |
| 5100000 | 5.76243 | 3746000 | 5.04445 | 3800000 | 3.78394 |
| 5150000 | 6.32159 | 3846000 | 5.89147 | 3900000 | 3.81104 |
| 5200000 | 6.38647 | 3946000 | 6.52755 | 4000000 | 3.85939 |
| 5250000 | 6.64571 | 4046000 | 7.57134 | 4100000 | 4.27538 |
| 5300000 | 7.55441 | 4146000 | 8.39647 | 4200000 | 4.67644 |
| 5350000 | 7.56904 | 4246000 | 9.5219 | 4300000 | 5.0775 |
| 5400000 | 8.53841 | 4296000 | 11.1072 | 4400000 | 5.08086 |
| 5450000 | 9.37901 | 4346000 | 11.955 | 4500000 | 5.31296 |
| 5500000 | 9.753 | 4396000 | 12.8939 | 4600000 | 5.55125 |
| 5520000 | 10.127 | 4426000 | 13.4174 | 4700000 | 5.95751 |
| 5540000 | 10.7449 | 4446000 | 13.9553 | 4800000 | 5.96103 |
| 5550000 | 11.1337 | 4476000 | 15.0963 | 4900000 | 5.97207 |
| 5575000 | 11.3227 | 4496000 | 15.7798 | 5000000 | 6.44329 |
| 5600000 | 11.6226 | 4521000 | 16.7856 | 5100000 | 6.7774 |
| 5625000 | 12.2515 | 4546000 | 18.8673 | 5200000 | 7.42116 |
| 5650000 | 12.9395 | 4571000 | 19.3336 | 5300000 | 7.58881 |
| 5675000 | 13.4817 | 4596000 | 21.0171 | 5400000 | 8.81037 |
| 5700000 | 14.5963 | 4621000 | 23.2129 | 5500000 | 10.2484 |
| 5725000 | 15.3215 | 4642000 | 28.1933 | 5600000 | 12.4828 |
| 5750000 | 16.7246 |  |  | 5700000 | 14.8272 |
| 5775000 | 17.8926 |  |  | 5750000 | 16.506 |
| 5800000 | 18.8303 |  |  | 5775000 | 17.5329 |
| 5825000 | 20.1069 |  |  | 5800000 | 19.0046 |
| 5835000 | 21.2511 |  |  | 5825000 | 20.2181 |
| 5845000 | 22.677 |  |  | 5850000 | 22.1729 |
| 5850000 | 23.4765 |  |  | 5875000 | 25.7207 |
| 5855000 | 24.987 |  |  |  |  |
| 5860000 | 26.3369 |  |  |  |  |
